# Feasibility of Lobectomy in Selected Patients with Unilateral N1b Papillary Thyroid Cancer

**DOI:** 10.1245/s10434-024-16643-5

**Published:** 2024-12-07

**Authors:** Danxia Li, Guojun Zhang, Xinna Li, Shuo Xu, Haiqing Sun, Yang Liu, Guochang Wu, Haitao Zheng, Xiaoli Zhang, Guibin Zheng

**Affiliations:** 1 Department of Thyroid Surgery, Yantai Yuhuangding Hospital, Qingdao University, Yantai, China; 2 Department of Breast and Thyroid Surgery, Changle People’s Hospital, Weifang, China; 3 Department of Pathology, Yantai Yuhuangding Hospital, Qingdao University, Yantai, China; 4 Department of Breast Surgery, Yantai Yuhuangding Hospital, Qingdao University, Yantai, China

**Keywords:** Lateral neck lymph nodes metastasis, Lobectomy, Papillary thyroid cancer, Recurrent-free survival, Surgical complications

## Abstract

**Background:**

Total thyroidectomy (TT) is usually recommended for unilateral papillary thyroid cancer (PTC) with lateral lymph node metastasis (LLNM), but no significant improvement in recurrence-free survival (RFS) is seen upon treatment. As an initial surgery, lobectomy may have advantages in appropriately selected unilateral PTC with ipsilateral LLNM.

**Objective:**

This study aimed to explore the feasibility of lobectomy for selected unilateral PTC with ipsilateral LLNM.

**Methods:**

From January 2014 to December 2021, we retrospectively reviewed patients with PTC and LLNM who were treated at our center. Patients preoperatively diagnosed with unilateral PTC and ipsilateral LLNM were recruited. Overall, 102 patients who chose lobectomy as their initial surgery were included in the lobectomy group and 96 patients who chose TT were included in the control group, defined as the TT group.

**Results:**

The mean follow-up time of the lobectomy group was 47.5 ± 22.2 months. Patients in the lobectomy group had a significantly lower rate of hypoparathyroidism than those in the TT group (0% vs. 11.5%; *p *< 0.001). RFS after lobectomy was comparable with that after TT according to Kaplan–Meier curve analysis (log-rank *p *= 0.80). Lobectomy achieved a significantly lower incidence of unsatisfactory TSH control than TT (5.9% vs. 20.8%; *p *= 0.006).

**Conclusions:**

Lobectomy may be an appropriate initial therapy for selected unilateral PTC with ipsilateral LLNM. A randomized prospective study with long-term follow-up is warranted.

**Supplementary Information:**

The online version contains supplementary material available at 10.1245/s10434-024-16643-5.

Papillary thyroid cancer (PTC) has been increasingly diagnosed and treated in recent decades.^1,2^ Although PTC is a relatively indolent tumor, it is usually accompanied by cervical lymph node metastasis at initial surgery. The incidence of PTC with lateral lymph node metastasis (LLNM) at initial surgery is 3.5–42%.^3–6^ When PTC metastasizes to the lateral compartment, it is classified as N1b, and therapeutic lateral neck dissection (LND) is needed. According to the 2015 American Thyroid Association (ATA) guidelines, total thyroidectomy (TT) is strongly recommended as the initial procedure for N1b PTC, regardless of unilateral or bilateral involvement.^7^

TT is used in unilateral PTC with LLNM mainly due to concerns about recurrence in the contralateral lobe and the postoperative application of radioactive iodine (RAI) ablation. However, according to the 2015 ATA guidelines, LLNM is not a risk factor in the stratification system for recurrence risk.^7^ Studies have shown that RAI treatment does not improve recurrence-free survival (RFS) and overall-survival in PTC;^8–10^ thus, the use of TT as an initial treatment for unilateral PTC with LLNM is controversial. Song et al. reported that RFS after lobectomy or TT was comparable in unilateral papillary thyroid microcarcinoma with ipsilateral LLNM.^11^ The same results were observed in patients with TNM stage T1 and T2 PTC with LLNM.^12^ Moreover, there is evidence that TT does not have a survival advantage over lobectomy.^13,14^ Lifelong thyroid-stimulating hormone (TSH) suppression by exogenous levothyroxine (L-T4) after TT may increase the risk of osteoporotic fractures and cardiovascular diseases.^15^ Thus, lobectomy may be justified in appropriately selected patients with unilateral PTC and ipsilateral LLNM.

In this study, we performed a single-center retrospective study to explore the feasibility of lobectomy in selected patients with unilateral PTC and ipsilateral LLNM, and evaluated the efficacy of lobectomy on the prognosis of these patients.

## Methods

### Patients and Study Design

This study was approved by the Ethics Committee of Yantai Yuhuangding Hospital (approval no. 2020-267). All patients included in the study were fully informed of the advantages and disadvantages of the surgical strategy preoperatively and subsequently signed informed consent forms. The informed consent for publication was waived by the Ethics Committee of our center as this was a retrospective study. Overall, 102 patients with PTC and LLNM who had undergone lobectomy at our center from January 2014 to December 2021 were included in the lobectomy group. Preoperative examinations such as thyroid function tests, ultrasonography (US), and enhanced computed tomography (CT) were routinely performed. Laryngoscopy was performed to assess vocal cord function before surgery. A multidisciplinary discussion (including a US expert and experienced radiologist) was performed preoperatively and candidates for lobectomy were determined. The inclusion criteria were (1) preoperative US identified unilateral suspicious nodules (Thyroid Imaging Reporting and Data System [TIRADS] ≥4b) and fine-needle aspiration (FNA) biopsy of confirmed or suspected PTC; (2) preoperative US suggested that no nodules or nodules ≤3 cm could be excised intraoperatively, or nodules (TIRADS ≤3) <1 cm that could be under active surveillance postoperatively in the contralateral gland; (3) preoperative imaging (US or CT) revealed suspected cervical lymph node metastasis in the ipsilateral lateral neck, which was confirmed by FNA or intraoperative frozen section biopsy; (4) no suspected lymph node metastasis was found in the contralateral central and lateral compartment by US and enhanced CT; and (5) the patient was willing to undergo lobectomy after being fully informed.

**Fig. 1:**
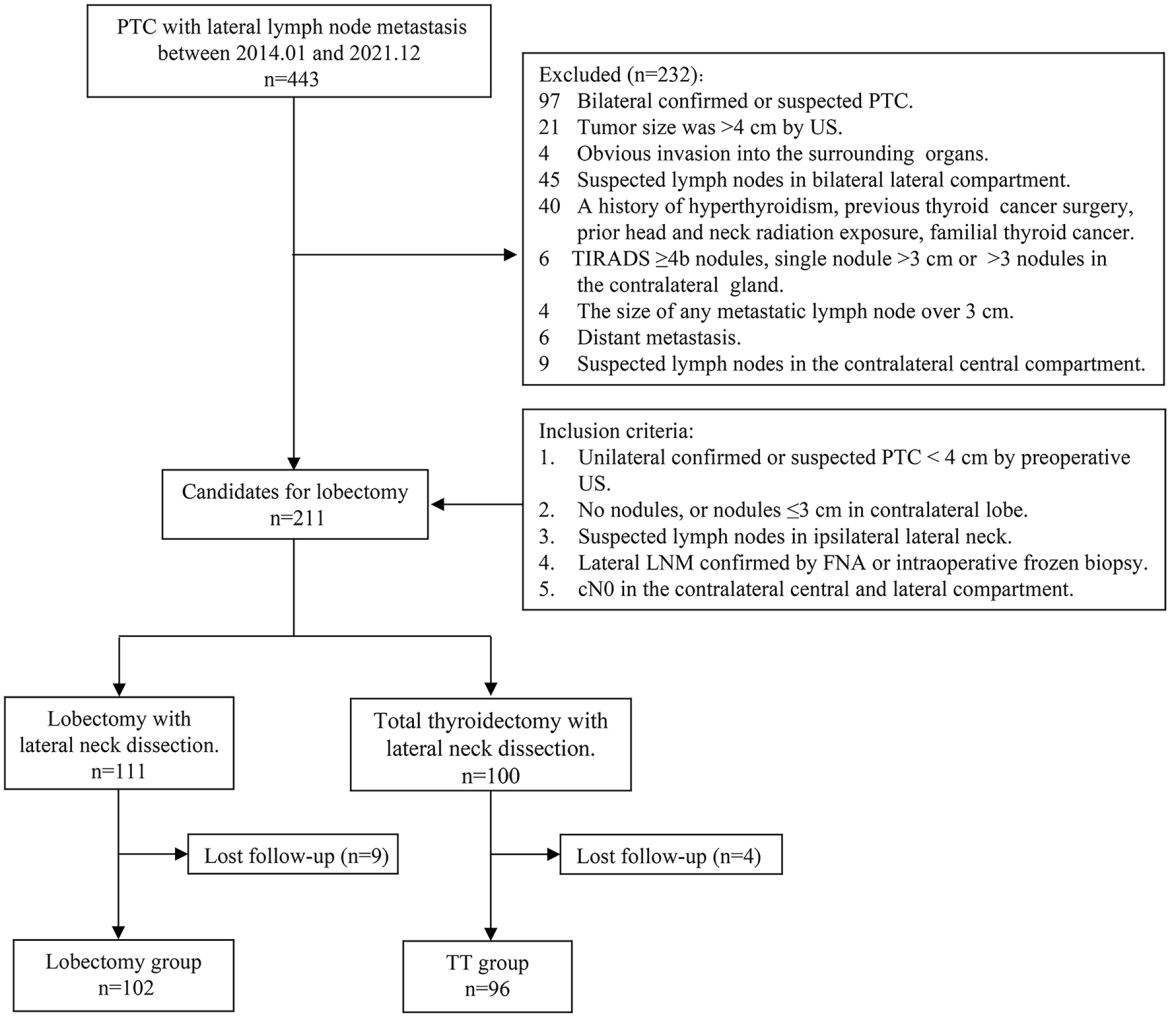
Inclusion and exclusion criteria for the lobectomy and total thyroidectomy groups. *PTC* papillary thyroid cancer, *US* ultrasonography, *LNM* lymph node metastasis, *FNA* fine needle aspiration, *TIRADS* thyroid imaging reporting and data system, *TT* total thyroidectomy

The exclusion criteria were (1) tumor size >4 cm on US; (2) preoperative imaging indicated that the tumor had invaded the surrounding organs, such as the trachea and esophagus; (3) preoperative US suggested that there were TRIADS ≥4b nodules in the contralateral gland without FNA, the maximum diameter of a single nodule was >3 cm in the contralateral gland, or there were more than three nodules; (4) preoperative imaging (US or CT) revealed the size of any metastatic lymph node was either over 3 cm in size or was fixed; (5) a history of hyperthyroidism, previous thyroid cancer surgery, prior head and neck radiation exposure, familial thyroid carcinoma, or contraindications for surgery; and (6) distant metastasis. The flowchart for patient enrollment in the two groups is shown in Fig. 1. Patients who had undergone lobectomy were enrolled in the lobectomy group. Ninety-six patients who met the inclusion criteria and who had undergone TT were included in the control group, defined as the TT group.

### Surgery Strategies

All surgeries were performed by experienced surgeons. TT or lobectomy was performed according to the preoperative surgical plan. All tumor foci were removed with R0 resection. Central neck dissection (CND), including the Delphian, pretracheal, and paratracheal lymph nodes, was routinely performed on the tumor side following thyroidectomy. The extent of LND normally includes the IIa, IIb, III, IV, and Vb regions. The minimum regions of the LND were IIa, III, and IV.

### Postoperative Management and Follow-Up

Complications of interest were hematoma, hypoparathyroidism, chyle leak, and nerve damage (the recurrent laryngeal nerve [RLN], accessory nerve, vagus nerve, phrenic nerve, and/or sympathetic trunk). Laryngoscopy was performed to evaluate vocal cord function. Transient RLN injury was defined as vocal cord paralysis that recovered within 6 months, while permanent RLN injury was defined as vocal cord paralysis lasting for >6 months. Serum parathyroid hormone (sPTH) levels were measured the day after surgery in patients who had undergone TT. Hypoparathyroidism was defined as an sPTH level below the lower limit (15.0 pg/mL), while permanent hypoparathyroidism was defined as a persistent sPTH level <15 pg/mL and/or the need for supplemental calcium and calcitriol for >6 months after surgery. L-T4 was prescribed for endocrine suppressive therapy according to the ATA guidelines.^7^ According to the histopathology results, RAI ablation was administered to some patients who had undergone TT.

Follow-up was usually performed at 1, 3, and 6 months, and every 6 months thereafter. Thyroid function tests were performed at every clinical visit, including thyroglobulin (Tg), thyroglobulin (anti-Tg) antibodies, and TSH. US was performed every 6 months after surgery. Unsatisfactory TSH control was defined as a TSH level not reaching the targeted level (according to the 2015 ATA guidelines) after L-T4 administration but with elevated free T3 (fT3) and/or free T4 (fT4) in a 12-month period. Enhanced CT was performed when suspected structural recurrence was detected using US. US-guided FNA was used for any suspected thyroid nodules or lymph nodes found on US. If persistently elevated serum Tg levels or structural recurrence were detected in the lobectomy group, lung CT, bone scanning, or positron emission tomography (PET) and CT were recommended to confirm whether or not distant metastasis was present. Iodine 131 whole-body scan or PET-CT was recommended for patients in the TT group without structural recurrence but with persistently elevated serum Tg levels.

### Statistical Analysis

SPSS version 20.0 (IBM Corporation, Armonk, NY, USA) and R software version 4.0.3 (The R Foundation for Statistical Computing, Vienna, Austria) were used for statistical analyses. Categorical variables were tested using the Chi-square test, Fisher’s exact test, or Kruskal–Wallis test, and continuous variables were analyzed using the Student’s t-test. RFS was estimated using the Kaplan–Meier method, and the curves were compared between groups, using log-rank tests. All statistics were two-sided and a *p*-value <0.05 was considered statistically significant.

## Results

### Demographics

From January 2014 to December 2021, 102 patients with LLNM who had undergone lobectomy were included in the lobectomy group, and 96 patients with LLNM who had undergone TT and met the inclusion criteria were included in the TT group (Table 1). No statistical differences were observed in sex, age, Hashimoto thyroiditis, tumor location, tumor size, multifocality, extrathyroidal extension (ETE), BRAF^V600^^E^ mutation, and histopathologic variants between the two groups. Central lymph node metastasis (CLNM) was observed in 87 patients (85.3%) in the lobectomy group and 85 patients (88.5%) in the TT group (*p *= 0.534). The number of central nodes, lateral nodes, CLNMs, and LLNMs removed were comparable between the two groups.

**Table 1:** Clinicopathologic characteristics of patients with PTC and lateral nodal metastasis

Variables	Lobectomy group [*n* = 102] (%)	TT group [*n* = 96] (%)	*p*-Value
Sex (female/male)	68/34	69/27	0.523^a^
Age, years (mean ± SD)	42.0 ± 12.7	43.3 ± 13.2	0.473^b^
Thyroiditis	19 (18.6)	29 (30.2)	0.083^a^
Tumor location			0.526^c^
Upper third	46 (45.1)	39 (40.6)	
Middle third	40 (39.2)	50 (52.1)	
Lower third	16 (15.7)	7 (7.3)	
Tumor size, cm (mean ± SD)	1.4 ± 0.8	1.6 ± 0.8	0.054^b^
Multifocality	17 (16.7)	26 (27.1)	0.109^a^
Capsular invasion	78 (76.5)	80 (83.3)	0.305^a^
ETE	14 (13.7)	13 (13.5)	1.000^a^
BRAF^V600E^ gene			0.089^c^
Mutant	58 (56.9)	50 (52.1)	
Wild	25 (24.5)	18 (18.8)	
Undetected	19 (18.6)	28 (29.2)	
Histopathologic variants			0.123^c^
Classical	71 (69.6)	54 (56.3)	
Follicular	18 (17.6)	32 (33.3)	
Tall cell	1 (1.0)	0	
Diffuse sclerosing	9 (8.8)	9 (9.4)	
Other	3 (2.9)	1 (1.0)	
Central LNM	87 (85.3)	85 (88.5)	0.534^a^
No. of central nodes removed (mean ± SD)	9.1 ± 4.5	9.9 ± 6.1	0.280^b^
No. of central LNMs (mean ± SD)	4.3 ± 3.5	4.51 ± 4.0	0.755^b^
No. of lateral nodes removed (mean ± SD)	25.3 ± 10.4	23.7 ± 8.9	0.257^b^
No. of lateral LNMs (mean ± SD)	4.0 ± 2.7	4.5 ± 2.4	0.176^b^
Size of the largest lymph node metastases, cm (mean ± SD)	0.76 ± 0.54	0.89 ± 0.52	0.073^a^

^a^Chi-square test

^b^Student’s t-test

^c^Kruskal–Wallis test

*PTC* papillary thyroid cancer, *ETE* extrathyroidal extension, *LNM* lymph nodes metastasis, *SD* standard deviation, *TT* total thyroidectomy

### Contralateral Lobe Comparisons

As shown in Table 2, no significant difference in the nodules of the contralateral lobe was observed between the two groups (*p *= 0.171). In the TT group, TIRADS 4a and 3 nodules in the contralateral lobe were found in 11 (11.4%) and 38 (39.6%) patients, respectively. Micro-PTC in the contralateral lobe was occasionally found in 10.6% (5/47) of patients without nodules, 54.5% (6/11) of patients with TIRADS 4a nodules, and 18.4% (7/38) of patients with TIRADS 3 nodules. In the lobectomy group, the nodules in eight patients were excised and pathologically confirmed as benign. During the follow-up period, 17.5% of patients (11/63) who had no nodules in the contralateral lobe at surgery were found to have new nodules in the contralateral lobe (electronic supplementary material [ESM] Table 1). Approximately 11.1% of patients (4/36) with TIRADS 3 nodules in the contralateral lobe developed TIRADS 4a or 4b nodules.

**Table 2:** Comparison of contralateral lobes between the lobectomy and TT groups

Variables		Lobectomy [*n* = 102]		TT [*n* = 96]		*P*
		Number (%)	Nodule size (cm) [range]	Number (%)	Nodule size (cm) [range]	
Nodule classification by TIRADS						0.171
	No nodule	63 (61.8)	–	47 (49.0)	–	
	4a	3 (2.9)^a^	0.9 ± 0.2 [0.8–1.1]	11 (11.4)	0.8 ± 0.7 [0.3–2.1]	
	≤3	36 (35.3)^b^	0.6 ± 0.3 [0.2–1.6]	38 (39.6)	0.7 ± 0.5 [0.2–2.0]	

PTC found in pathology			Tumor size (cm) [range]		Tumor size (cm) [range]	
	No nodule	–	–	5 (5.2)	0.14 ± 0.09 [0.1–0.3]	–
	4a	–	–	6 (6.3)	0.22 ± 0.10 [0.1–0.3]	
	≤3	3 (2.9)^c^	0.5 ± 0.3 [0.3–0.8]	7 (7.3)	0.23 ± 0.08 [0.2–0.4]	

^a^Nodules were removed and confirmed benign by pathology

^b^Nodules in five patients were removed and confirmed benign by pathology

^c^Three patients were confirmed to have PTC, by fine-needle aspiration, and received reoperations during the follow-up period

*PTC* papillary thyroid cancer, *TIRADS* thyroid imaging reporting and data system, *TT* total thyroidectomy

### Complications

There was no statistically significant difference in transient and permanent RLN injuries between the two groups. The RLN was invaded and sacrificed in six patients in the lobectomy (5.9%) group and eight patients in the TT group (8.3%). Although six patients in the lobectomy group had RLN invasion, no surgical plans were changed, because R0 resection was achieved in those patients. Iatrogenic RLN injury occurred in one patient (1.0%) in the TT group. Transient hypoparathyroidism occurred in 11 patients (11.5%), and permanent hypoparathyroidism occurred in one patient (1.0%) in the TT group. No transient or permanent hypoparathyroidism occurred in the lobectomy group (*p *< 0.001). Furthermore, there was a similar incidence of surgical complications in both groups with respect to hemorrhage, chylous fistula, and other nerve injuries (Table 3).

**Table 3:** Surgical complications between the lobectomy and TT groups

	Lobectomy [*n* = 102] (%)	TT [*n* = 96] (%)	*p*-Value
Hemorrhage	1 (1.0)	0	1.000^a^
RLN injury			0.511^b^
Transient	2 (2.0)	1 (1.0)	
Permanent	6 (5.9)^c^	9 (9.4)^d^	
Hypoparathyroidism			<0.001^b^
Transient	0	11 (11.5)	
Permanent	0	1 (1.0)	
Chylous fistula	3 (2.9)	2 (2.1)	1.000^a^
Accessory nerve	2 (2.0)	1 (1.0)	1.000^a^
Hypoglossal nerve	1 (1.0)	0	1.000^a^

^a^Fisher’s exact test

^b^Kruskal–Wallis test

^c^RLN was invaded by tumor in six cases

^d^RLN was invaded by tumor in eight cases

*TT* total thyroidectomy, *RLN* recurrent laryngeal nerve

### Follow-Up

The follow-up times for the lobectomy and TT groups were 47.5 ± 22.2 months and 52.0 ± 25.3 months, respectively (*p* = 0.185). As shown in Table 4, 78.1% of patients in the TT group received RAI treatment. The stimulated Tg level before the first RAI treatment was 13.1 ± 43.2 ng/mL, and the suppressed Tg level at the last follow-up was 0.5 ± 1.1 ng/mL. Tg >1 ng/mL at the last follow-up occurred in 10 patients (9.6%) in the TT group, with a mean Tg of 2.3 ± 2.0 ng/mL (range 1.05–7.19 ng/mL). Three patients (2.9%) in the lobectomy group underwent reoperations for recurrence in the contralateral lobe (ESM Table 2). The rate of unsatisfactory TSH control in the TT group was 20.8%, which was significantly higher than the rate of 5.9% in the lobectomy group (*p *= 0.006).

**Table 4:** Follow-up of the TT and lobectomy groups

Variables	TT [*n* = 96]	Lobectomy [*n* = 102]	*p*-Value
Follow-up time, months (mean ± SD) [range]	52.0 ± 25.3 [8–118]	47.5 ± 22.2 [12–114]	0.185^a^
RAI treatment	75 (78.1%)	0	<0.001^b^
sTg before first RAI (ng/mL)^d,e^	13.1 ± 43.2 [0.04–313.4]	–	–
Suppressed Tg at last follow-up (ng/mL)^d,e^	0.5 ± 1.1 [0.04–7.2]	–	–
Unsatisfactory TSH control	20 (20.8%)	6 (5.9%)	0.006^c^
Recurrence	5 (5.2%)	6 (5.9%)	1.000^b^

^a^Student’s t-test

^b^Fisher’s exact test

^c^Chi-square test

^d^Seven patients with elevated TgAb were excluded

^e^Tg was considered as 0.04 in order to calculate it when it was undetectable (<0.04)

*TT* total thyroidectomy, *RAI* radioactive iodine, *SD* standard deviation, *Tg* thyroglobulin, *sTg* stimulated thyroglobulin, *TSH* thyroid-stimulating control, *TgAb* antithyroglobulin antibody

**Fig. 2:**
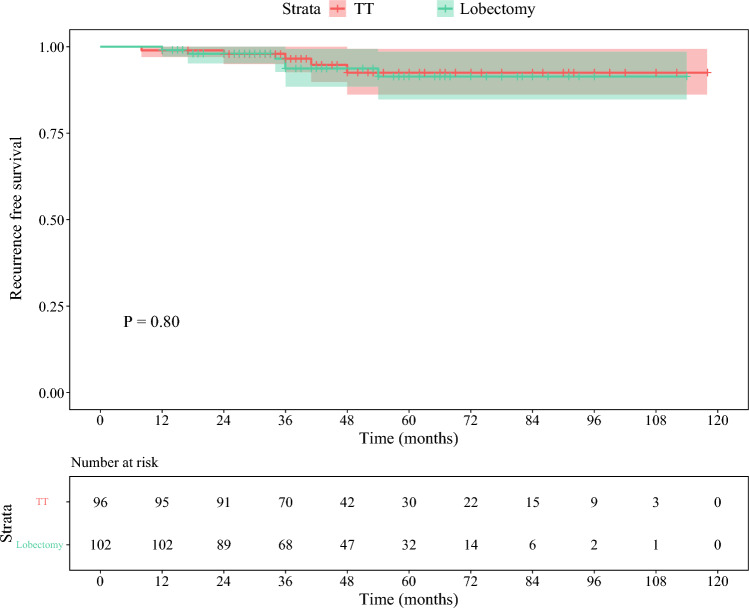
Kaplan–Meier survival curve for recurrence-free survival in the lobectomy and TT groups. *TT* total thyroidectomy

Six patients (5.9%) in the lobectomy group and five patients (5.2%) in the TT group experienced recurrence and underwent reoperation during the follow-up period (ESM Table 2). No statistically significant difference in recurrence rate was observed between the two groups (log-rank *p* = 0.80) (Fig. 2).

## Discussion

In the United States, the rate of TT for PTC increased every year from the years 2000 to 2014, and TT was more likely to be performed in patients with PTC with tumor sizes <4 cm;^16^ however, multiple studies have shown that TT did not contribute to reduced recurrence and survival benefits.^11–14,17^ Our study showed that the efficacy of lobectomy for RFS was comparable with that of TT (log-rank *p* = 0.80) (Fig. 2) in selected unilateral PTC with ipsilateral LLNM. Moreover, lobectomy could lower the rate of unsatisfactory TSH control.

Wang et al.^12^ and Song et al.^11^ reported equivalent RFS between lobectomy and TT in appropriately selected patients with unilateral PTC and LLNM; however, the condition of the contralateral lobe has not yet been reported. Dong et al.^18^ reported that the recurrence rate of PTC in the contralateral remnant thyroid tissue was 1.5% (7/466) over a median 18.4-year follow-up period. In our study, occasional minimal PTC was found in the contralateral lobe in approximately 18.8% of patients (18/96) in the TT group. Only 2.9% of patients (3/102) in the lobectomy group had reoperations for confirmed PTC in the contralateral lobe, which is consistent with the study by Dong et al.^18^ Although our follow-up period was relatively short, it covered the peak time of recurrence (1–3 years) of PTC, as reported in previous literature.^19–23^ Therefore, we concluded that lobectomy did not increase the risk of reoperation of the contralateral lobe within a relatively short follow-up period. It should be noted that minimal PTC was detected in approximately 54.5% of patients (6/11) in the TT group who had TIRADS 4a nodules in the contralateral lobe; therefore, TT should be recommended to these patients to reduce possible recurrence.

It was reported that ETE was an important risk factor for recurrence in patients with LLNM.^22,24^ Although 6% of patients in the lobectomy group exhibited ETE, the multicenter study by Kuba et al.^25^ reported that the 10-year RFS after TT was comparable with that after lobectomy. Our study included 13.7% of patients with ETE in the lobectomy group, and we found no significant difference in RFS between the lobectomy and TT groups. Evidence has also shown that there are no differences in overall survival (OS) or disease-specific survival after TT or lobectomy, even in patients who had undergone lobectomy accompanied by high-risk factors, including ETE.^14,17^ Thus, lobectomy may be an alternative strategy for patients with unilateral PTC and LLNM carrying high-risk factors such as RLN invasion.

There is no doubt that lobectomy is better tolerated than TT, particularly for parathyroid protection. Kuba et al.^25^ reported that the incidence rates of transient and permanent hypocalcemia after TT were 38% and 8%, respectively, a result that was similar to that reported by Song et al. (43.3% and 4.5%, respectively).^11^ In our study, the incidence of transient and permanent hypocalcemia in the TT group was 11.5% and 1.0%, respectively. Due to the exclusion of PTC with ETE, no permanent RLN injury occurred in the studies by Song et al.^11^ and Wang et al.^12^ Kuba et al.^25^ reported that the incidence of RLN paralysis over a 1-year period was not significantly different between the TT and lobectomy groups (7.5% vs. 2.5%; *p *= 0.203). In our study, the incidence rates of permanent RLN injury in the lobectomy and TT groups were 5.9% and 9.4% respectively; however, approximately 93.3% of RLN injuries (14/15) were due to tumor invasion, and in these patients, the RLN was excised to achieve R0 resection. Given the similar rates of RFS between the two groups in our study, lobectomy was considered an alternative option when the RLN was invaded. This approach could reduce the risk of tracheotomy caused by bilateral RLN injury.

RAI therapy is usually recommended for patients with LLNM after TT and could contribute to recurrence detection using Tg; however, there is evidence showing that it did not improve RFS and OS.^8–12,25^ In our study, the recurrence rate for patients who received RAI treatment was comparable with that of patients who did not receive RAI treatment in the TT group (5.3% vs. 4.8%; *p* = 1.000). In order to fully explore the benefits of RAI treatment following TT, patients who did not receive RAI treatment in the TT group were excluded (ESM Table 3). However, in our cohort, we did not observe a significant improvement in RFS as a result of RAI therapy following TT (ESM Fig. 1). Thus, RAI therapy should not be the definitive reason for performing TT in some patients with unilateral PTC and ipsilateral LLNM.

Exogenous L-T4 intake for TSH suppression is an important postoperative therapy for patients with PTC; however, TSH suppression could increase the risk of coronary heart disease, ischemic stroke, reduced bone mineral density, and osteoporotic fractures,^15,26,27^ which are closely related to the dose of L-T4. Wang et al.^12^ reported that compared with TT, lobectomy in properly selected PTC with LLNM could significantly reduce the intake dose of L-T4. Thus, cardiovascular changes, especially an increased resting heart rate, were significantly reduced in patients who had undergone lobectomy. A multicenter prospective study that included 2013 patients with differentiated thyroid cancer indicated that 1236 patients (61.4%) achieved targeted values of TSH suppression at 1 year after surgery.^28^ A prospective study by Chen et al.^29^ showed that the incidence of well-controlled TSH levels 1 year after lobectomy was significantly higher than that after TT. Our study showed a lower rate of unsatisfactory TSH control in patients who had undergone lobectomy. Therefore, lobectomy could reduce anxiety caused by unsatisfactory TSH control and the potential risk of cardiovascular disease and osteoporotic fractures.

Our study had several limitations. First, the follow-up duration in our study was relatively short; therefore, a study with a longer time period is needed to further illustrate the outcome of lobectomy in unilateral N1b PTC. Second, the benefit to the cardiovascular and skeletal organs was not evaluated because of the lack of related data collection. Third, although the candidates for lobectomy were carefully selected by a multidisciplinary team before surgery based on strict enrollment criteria, there was a selection bias among patients in the lobectomy group, which may be due to the influence of the consulting surgeons. Thus, a multicenter, randomized, prospective study is needed to further illustrate the oncological safety of lobectomy in these patients.

## Conclusion

Lobectomy achieved a comparable RFS compared with TT, and lowered unsatisfactory TSH control in properly selected patients with unilateral PTC and ipsilateral LLNM. Moreover, lobectomy had a significant advantage over TT in reducing the incidence of hypoparathyroidism. Thus, lobectomy may be an alternative option for properly selected patients with unilateral PTC and ipsilateral LLNM. A prospective, randomized, controlled trial across multiple centers with long-term follow-up is needed to further confirm the efficacy and outcomes of lobectomy.

## Supplementary Information

Below is the link to the electronic supplementary material.

Supplementary file1 (DOCX 81 KB)

## Data Availability

The data that support the findings of this study are available from the corresponding author upon reasonable request.
