# Supplementary material for: Feasibility of Lobectomy in Selected Patients with Unilateral N1b Papillary Thyroid Cancer
Source: Ann Surg Oncol. 2024 Dec 7;32(4):2344–52. doi: 10.1245/s10434-024-16643-5 (PMC11882689; doi:10.1245/s10434-024-16643-5)
Supplement: Supplementary file 1 — Supplementary file1 (DOCX 81 KB) [file 10434_2024_16643_MOESM1_ESM.docx]

**Supplementary tables and figure- Index**

| **Supplementary Tables** |  |
| --- | --- |
| Table 1 | *pag. 2* |
| Table 2 | *pag. 3* |
| Table 3 | *pag. 4* |
| **Supplementary Figure** |  |
| Figure 1 | *pag. 5* |

| **Supplementary Table 1. Follow-up of the contralateral lobes in the lobectomy group.** | | | | |
| --- | --- | --- | --- | --- |
|  | New nodules or nodules progression | | | Treatment |
|  | Classification by TIRADS | N | size (cm) [range] |  |
| No nodule (n=63) |  |  |  |  |
|  | 4a | 1 | 0.4 | - |
|  | ≤3 | 10 | 0.36±0.10  [0.2-0.5] | - |
| 4a (n=3)^*^ | ≤3 | 1 | 0.5 | - |
| ≤3 (n=36)^†^ |  |  |  | - |
|  | 4b | 1 | 0.8 | Re-operation |
|  | 4a | 3 | 0.43±0.12  [0.3-0.5] | Re-operation in 2 patients^‡.^ |
| ^*^ The nodules were removed and confirmed benign by pathology at the primary surgery.  ^†^ The nodules in 5 patients were removed and confirmed benign by pathology at the primary surgery.  ^‡^ 2 patients were performed re-operation for PTC in the contralateral lobe confirmed by FNA and 1 patients was under active surveillance. | | | | |

| **Supplementary Table 2. Detailed information of the recurrence cases in the lobectomy and TT groups** | | | | | | |
| --- | --- | --- | --- | --- | --- | --- |
| Cases | Relapse time  (months) | Histopathologic variants | Recurrence site | Reoperation range | Pathology | |
|  |  |  |  |  | Removed lobe (cm) | Removed lymph nodes |
| Lobectomy group | |  | |  |  |  |
| 1 | 17 | Classical variant | Contralateral lobe | Contralateral lobectomy + CND | PTC, 0.8 + 0.2 | CC: 0/6 |
| 2 | 54 | Follicular variant | Contralateral lobe | Contralateral lobectomy + CND | PTC, 0.3 | CC: 1/6 |
| 3 | 34 | Classical variant | Ipsilateral lateral neck | Salvage LND + CND | - | LC: 1/6  CC: 0/4 |
| 4 | 46 | Follicular variant | Contralateral lateral neck | Contralateral lobectomy + CND + LND | Benign | LC: 2/22  CC: 0/6 |
| 5 | 12 | Classical variant | Contralateral central neck | Contralateral lobectomy + CND | Benign | CC: 1/5 |
| 6 | 36 | Classical variant | Contralateral lobe | Contralateral lobectomy + CND | PTC, 0.5 | CC: 0/4 |
| TT group | |  | |  |  |  |
| 1 | 48 | Follicular variant | Ipsilateral lateral neck | Salvage LND | - | LC: 2/21 |
| 2 | 8 | Classical variant | Contralateral lateral neck | Contralateral LND | - | LC: 1/28 |
| 3 | 24 | Follicular variant | Ipsilateral central neck | Salvage CND | - | CC: 1/4 |
| 4 | 41 | Classical variant | Ipsilateral central neck | Salvage CND | - | CC: 2/6 |
| 5 | 36 | Classical variant | Ipsilateral lateral neck | Salvage LND | - | LC: 2/10 |
| LND, lateral neck dissection; CND, central neck dissection; LC, lateral compartment; CC, central compartment. TT, total thyroidectomy. | | | | | | |

| **Supplementary Table 3. Clinicopathologic characteristics of patients in the lobectomy group and patients in the TT group who undergone I^131^ treatment before and after PSM.** | | | | | | | |
| --- | --- | --- | --- | --- | --- | --- | --- |
| Variables | Before PSM | | |  | After PSM | | |
|  | Lobectomy  (n=102) (%) | TT  (n=75) (%) | *P* |  | Lobectomy  (n=55) (%) | TT  (n=55) (%) | *P* |
| Sex (female/male) | 68/34 | 55/20 | 0.431^*^ |  | 38/17 | 40/15 | 0.834^*^ |
| Age, years (mean ± SD) | 42.0 ± 12.7 | 42.0 ± 12.6 | 0.985^†^ |  | 42.4 ±13.3 | 41.8 ±13.6 | 0.799^†^ |
| Thyroiditis | 19 (18.6) | 21 (28.0) | 0.197^*^ |  | 14 (25.5) | 12 (21.8) | 0.822^*^ |
| Tumor location |  |  | 0.748^‡^ |  |  |  | 0.843^‡^ |
| Upper 1/3 | 46 (45.1) | 32 (42.7) |  |  | 19 (34.5) | 20 (36.4) |  |
| Middle 1/3 | 40 (39.2) | 41 (54.7) |  |  | 28 (50.9) | 33 (60.0) |  |
| Lower 1/3 | 16 (15.7) | 2 ( 2.7) |  |  | 8 (14.5) | 2 ( 3.6) |  |
| Tumor size, cm (mean ± SD) | 1.4 ± 0.8 | 1.6 ± 0.7 | 0.062^†^ |  | 1.5 ±0.7 | 1.5 ±0.7 | 0.761^†^ |
| Multifocality | 17 (16.7) | 21 (28.0) | 0.103^*^ |  | 11 (20.0) | 12 (21.8) | 1.000^*^ |
| Capsular invasion | 78 (76.5) | 62 (82.7) | 0.415^*^ |  | 43 (78.2) | 46 (83.6) | 0.628^*^ |
| ETE | 14 (13.7) | 12 (16.0) | 0.836^*^ |  | 10 (18.2) | 9 (16.4) | 1.000^*^ |
| BRAF^V600E^ gene |  |  | 0.276^‡^ |  |  |  | 0.696^‡^ |
| Mutant | 58 (56.9) | 39 (52.0) |  |  | 25 (45.5) | 30 (54.5) |  |
| Wild | 25 (24.5) | 16 (21.3) |  |  | 15 (27.3) | 14 (25.5) |  |
| Undetected | 19 (18.6) | 20 (26.7) |  |  | 15 (27.3) | 11 (20.0) |  |
| No. of central nodes removed (mean ± SD) | 9.1 ± 4.5 | 10.6 ± 6.2 | 0.056^†^ |  | 9.5 ±4.7 | 10.1 ±5.5 | 0.503^†^ |
| No. of central LNM  (mean ± SD) | 4.3 ± 3.5 | 5.0 ± 4.1 | 0.285^†^ |  | 4.6 ±3.5 | 4.8 ±3.7 | 0.792^†^ |
| No. of lateral nodes removed (mean ± SD) | 25.3 ± 10.4 | 24.6 ± 9.2 | 0.634^†^ |  | 25.5 ±10.3 | 24.9 ±9.0 | 0.746^†^ |
| No. of lateral LNM  (mean ± SD) | 4.0 ± 2.7 | 4.9 ± 2.4 | 0.026^†^ |  | 3.9 ±2.6 | 4.8 ±2.4 | 0.065^†^ |
| ^*^Chi-square test; ^†^Student’s t-test; ^‡^Kruskal–Wallis test  ETE, extrathyroidal extension; LNM, lymph nodes metastasis;PSM,propensity score matching; SD, standard deviation; TT, total thyroidectomy. | | | | | | | |

**Supplementary Figure**

**
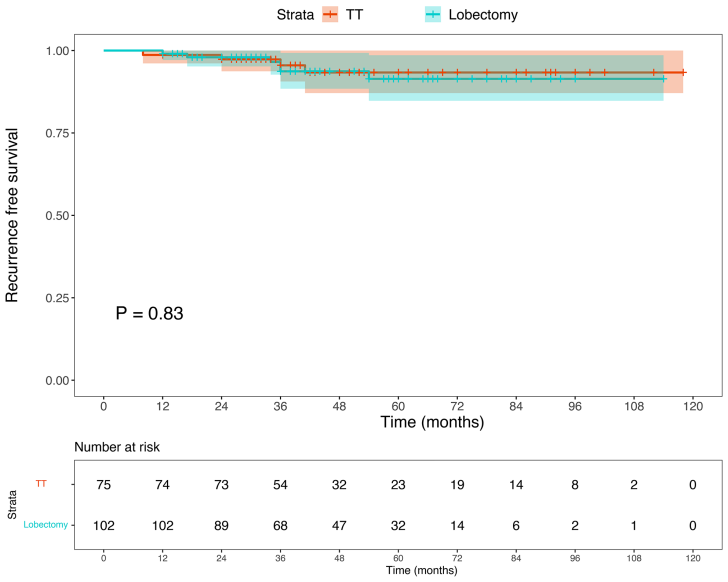
**

**Supplementary Figure 1.** Kaplan–Meier survival curves for recurrence-free survival of the patients in the lobectomy group and patients who undergone radioactive iodine treatment in the TT groups.
